# Cell-Nonautonomous Regulation of *C. elegans* Germ Cell Death by *kri-1*

**DOI:** 10.1016/j.cub.2009.12.032

**Published:** 2010-02-23

**Authors:** Shu Ito, Sebastian Greiss, Anton Gartner, W. Brent Derry

**Affiliations:** 1 Developmental & Stem Cell Biology, The Hospital for Sick Children, Toronto, ON M5G 1X8, Canada; 2 Department of Molecular Genetics, University of Toronto, Toronto, ON M5S 1A8, Canada; 3 Wellcome Trust Centre for Gene Regulation and Expression, University of Dundee, Dundee DD1 5EH, UK

**Keywords:** DEVBIO, CELLCYCLE

## Abstract

Programmed cell death (or apoptosis) is an evolutionarily conserved, genetically controlled suicide mechanism for cells that, when deregulated, can lead to developmental defects, cancers, and degenerative diseases [1, 2]. In *C. elegans*, DNA damage induces germ cell death by signaling through *cep-1*/*p53*, ultimately leading to the activation of CED-3/caspase [3–13]. It has been hypothesized that the major regulatory events controlling cell death occur by cell-autonomous mechanisms, that is, within the dying cell. In support of this, genetic studies in *C. elegans* have shown that the core apoptosis pathway genes *ced-4*/*APAF-1* and *ced-3*/caspase are required in cells fated to die [9]. However, it is not known whether the upstream signals that activate apoptosis function in a cell-autonomous manner. Here we show that *kri-1*, an ortholog of *KRIT1*/*CCM1*, which is mutated in the human neurovascular disease cerebral cavernous malformation [14, 15], is required to activate DNA damage-dependent cell death independently of *cep-1*/*p53*. Interestingly, we find that *kri-1* regulates cell death in a cell-nonautonomous manner, revealing a novel regulatory role for nondying cells in eliciting cell death in response to DNA damage.

## Highlights

► *kri-1* is a novel positive regulator of *C. elegans* germ cell apoptosis ► *kri-1* regulates cell death independently of *cep-1*/*p53* ► *kri-1* regulates cell death cell nonautonomously, possibly by cross-tissue signaling

## Results and Discussion

**Figure 1:**
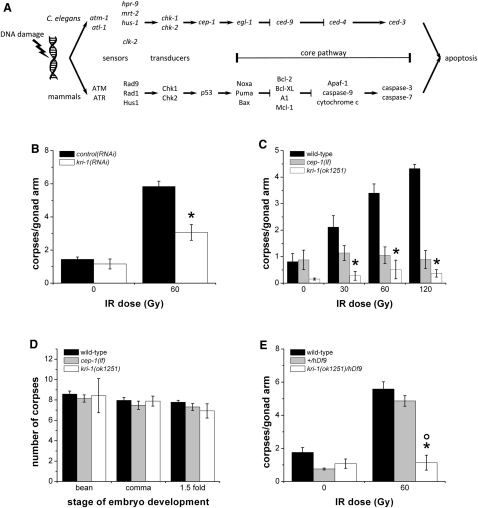
*kri-1* Is Required for DNA Damage-Induced Germ Cell Death Specifically (A) The conserved genetic pathway regulating cell death in mammals and *C. elegans*. Sensors and transducers relay DNA damage signals to CEP-1/p53, which transcriptionally activates one or more BH3-only genes to promote apoptosis. (B) Wild-type animals fed *control(RNAi)* (black) or *kri-1(RNAi)* (white) were subjected to ionizing radiation (IR) at 20°C, and germ cell apoptosis was quantified 24 hr later. Data represent mean ± standard error of the mean (SEM) of three independent experiments and at least 55 germlines in total per strain per condition. ^∗^p < 0.05 versus wild-type. See Table S1 for full list of p values. (C) Synchronized wild-type (black), *cep-1(lf)* (gray), and *kri-1(ok1251)* (white) young adult animals were treated with increasing doses of IR, and germ cell apoptosis was scored as above. Data represent mean ± SEM of at least three independent experiments and at least 50 germlines in total per strain per condition. ^∗^p < 0.05 versus wild-type. (D) Embryos at the indicated developmental stages were scored for apoptosis with Nomarski optics in wild-type (black), *cep-1(lf)* (gray bars), and *kri-1(ok1251)* (white) animals. Data represent mean ± SEM of three independent experiments and at least 45 embryos in total per strain per stage. (E) Apoptosis was scored in wild-type animals (black), a strain with a wild-type copy of *kri-1* in *trans* to the *hDf9* deficiency (gray), and *ok1251* in *trans* to *hDf9* (white) treated with IR as above. Data represent mean ± SEM of at least four independent experiments and at least 25 germlines in total per strain per condition. ^∗^p < 0.05 versus wild-type; °p < 0.01 between *kri-1(ok1251)/hDf9* and *+/hDf9*. See also Figure S1.

In an RNA interference (RNAi) screen unrelated to apoptosis, we serendipitously uncovered a *cep-1*/*p53*-interacting gene, *kri-1*, the ortholog of human *KRIT1*/*CCM1*, which is frequently mutated in the neurovascular disease cerebral cavernous malformation [14, 15]. Because this gene had been previously shown to integrate signals from reproductive tissues (germ cells) to elicit longevity effects in nonreproductive (somatic) tissues [16] and interacts with *cep-1*, an important mediator of germ cell death (Figure 1A) [7, 8], we asked whether *kri-1* is involved in a novel, cell-nonautonomous mechanism to regulate germ cell death. To test this, we first investigated whether *kri-1* regulates cell death like *cep-1*, by quantifying the number of germ cell corpses in wild-type animals fed bacteria producing double-stranded RNA against a control gene or *kri-1* exposed to ionizing radiation (IR) (Figure 1B). We found that knockdown of *kri-1* by RNAi significantly reduced the number of germ cell corpses after DNA damage (IR) compared to animals fed control RNAi (p = 0.01), suggesting that *kri-1* is required for germ cell death. We verified this initial observation by performing a dose-response analysis of the *kri-1(ok1251)* deletion mutant. In contrast to wild-type animals, *kri-1(ok1251)* deletion mutants did not exhibit an increase in germ cell apoptosis after exposure to increasing doses of IR (Figure 1C; see also Figure S1 available online). This was reminiscent of *cep-1* loss-of-function (lf) mutants that are also resistant to IR-induced apoptosis. Therefore, we examined whether *kri-1* regulates germ cell death specifically, like *cep-1*, or whether it regulates cell death in all cells, like *ced-3*, by quantifying apoptosis in developing embryos of wild-type animals and *cep-1(lf)* and *kri-1(ok1251)* mutants. We found that developmental cell death was unaffected in *kri-1(ok1251)* mutants, suggesting that the regulation of cell death by *kri-1* is specific to germ cells, like *cep-1* (Figure 1D). Finally, to determine whether the *ok1251* allele is a null, we performed a deficiency analysis by crossing *ok1251* into a strain containing the *hDf9* deficiency that removes the *kri-1* locus and quantified the number of germ cell corpses after DNA damage (Figure 1E). Strains containing the *ok1251* allele in *trans* to *hDf9* were as resistant to damage-induced germ cell apoptosis as *ok1251* homozygotes, suggesting that *ok1251* is a null allele. Collectively, these and further observations (see below) indicate that *kri-1* is specifically required for germ cell death in response to DNA damage.

**Figure 2:**
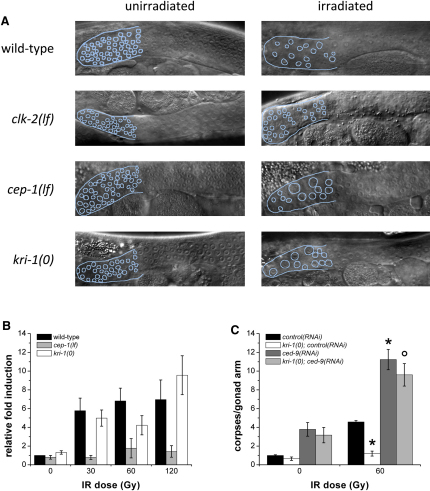
*kri-1* Functions Downstream of the Checkpoint Genes but Upstream of *ced-9* (A) Synchronized hermaphrodites at the fourth larval stage (L4) were treated with IR, and the number of nuclei per unit area in the mitotic region of the germline was quantified 24 hr later at 20°C. The mitotic region and nuclei have been outlined for clarity. Representative images from three independent experiments are shown. (B) RNA was isolated by TRIzol from synchronized wild-type (black), *cep-1(lf)* (gray), and *kri-1(0)* (white) mutants, and *egl-1* transcript levels were measured by quantitative real-time PCR. Data represent mean ± SEM of three independent experiments. (C) Synchronized wild-type and *kri-1(0)* L4 animals fed *control(RNAi)* (*Y95B8A_84.g*, a nonexpressed gene) (black and white, respectively) or *ced-9(RNAi)* (dark gray and light gray, respectively) were subjected to IR, and germ cell death was quantified as described above. Data represent mean ± SEM of three independent experiments and at least 25 germlines in total per strain per condition. ^∗^p < 0.01 versus wild-type; °p < 0.05 versus *kri-1(0); control(RNAi)*. See also Figure S2.

Given that *kri-1* is required to promote germ cell death in response to DNA damage, we were interested to know at which step in the pathway it might be functioning (Figure 1A). In the *C. elegans* germline, the DNA damage checkpoint genes (*hpr-9*, *mrt-2*, *hus-1*, and *clk-2*) are required to both transiently arrest mitotic proliferation and activate *cep-1*-dependent apoptosis of damaged germ cells [5, 6]. To ascertain whether *kri-1* is functioning in an analogous manner (i.e., upstream of *cep-1*), we tested whether *kri-1* null (0) mutants mimic the germline phenotypes of checkpoint gene mutants. In contrast to *clk-2* mutants that are defective in cell-cycle arrest, we found that *kri-1* was not required for IR-induced arrest of mitotically proliferating cells (Figure 2A; Figure S2A), implying that *kri-1* acts downstream or independently of the DNA damage checkpoint. To delineate whether *kri-1* is required to transduce signals to the CEP-1 protein and therefore allow apoptosis to occur, we examined the activity of CEP-1 by quantifying the transcript levels *egl-1*, a proapoptotic target gene of CEP-1 [17, 18]. Consistent with previous work, *egl-1* transcript levels as assessed by real-time quantitative PCR (qPCR) increased in response to DNA damage in wild-type animals, but not in *cep-1(lf)* mutants (Figure 2B). Interestingly, *egl-1* induction in *kri-1(0)* mutants was similar to that seen in wild-type animals, indicating that the transcriptional activity of CEP-1 is induced normally in the absence of *kri-1*. This is consistent with *kri-1* promoting damage-induced apoptosis downstream or independently of *cep-1*. Such a model raised the possibility that *cep-1* might regulate *kri-1* transcription or KRI-1 protein localization in response to DNA damage and that this was required to promote germ cell death. However, neither *kri-1* transcript levels nor GFP::KRI-1 localization was significantly affected by IR or *cep-1* status (Figures S2B–S2D).

The data above suggest a model wherein *kri-1* functions downstream of or in parallel to the key decision-making step in the cell death pathway and likely regulates components of the core death pathway (i.e., *egl-1*, *ced-9*, *ced-4*, and *ced-3*). To investigate this further, we examined the epistatic relationship between *kri-1* and *ced-9*. Healthy cells require functional CED-9/BCL2 to prevent ectopic activation of CED-3/caspase by CED-4 (Figure 1A). We reasoned that if *kri-1* functions downstream of *ced-9*, ablation of *kri-1* would suppress the increased cell death caused by *ced-9(lf)*; on the other hand, the converse would be true if *kri-1* acts upstream of *ced-9*. Knockdown of *ced-9* by RNAi (>50% knockdown; Figure S2E) caused a significant increase in apoptosis both before and after DNA damage, but this was unaffected by loss of *kri-1* (Figure 2C), which we confirmed in *kri-1(0); ced-9(lf)* double mutants (data not shown). This indicates that *kri-1* is not functioning strictly downstream of *ced-9* (i.e., in a manner similar to *ced-4* or *ced-3*). To be sure, we quantified the mRNA of both *ced-4* and *ced-3* by qPCR and found that their levels were not affected in *kri-1(0)* mutants in response to IR (Figures S2F–S2H); in addition, CED-4 protein expression and localization was not affected in *kri-1(0)* mutants (data not shown). Therefore, we infer from these results that *kri-1* acts upstream of, or parallel to, *ced-9*.

**Figure 3:**
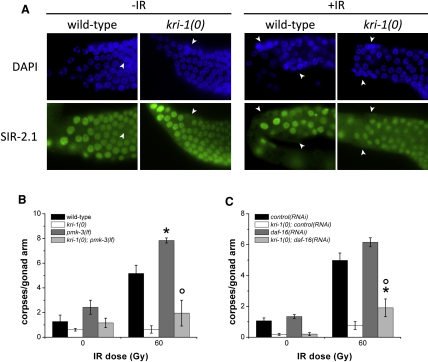
*kri-1* Functions Independently of Known *cep-1*-Independent Pathways (A) Wild-type and *kri-1(0)* animals were immunostained with DAPI or SIR-2.1 antibodies before and after IR. The images show the pachytene region of the germline. Arrowheads indicate nuclei positive for DAPI staining but negative for SIR-2.1 protein expression. Representative images of at least three independent experiments are shown. (B) Wild-type (black), *kri-1(0)* (white), *pmk-3(lf)* (dark gray), and *kri-1(0); pmk-3(lf)* (light gray) animals were synchronized and scored for apoptosis as described in Figure 1B. Data represent mean ± SEM of three independent experiments and at least 50 germlines in total per strain per condition. ^∗^p < 0.05 versus wild-type; °p < 0.05 versus *pmk-3(lf)*. (C) Germline apoptosis was quantified in synchronized wild-type and *kri-1(0)* mutants fed *control(RNAi)* (black and white, respectively) or *daf-16(RNAi)* (dark and light gray, respectively) and treated with IR as described above. Data represent mean ± SEM of four independent experiments and at least 25 germlines in total per strain per condition. ^∗^p < 0.01 versus wild-type; °p < 0.01 versus *daf-16(RNAi)*. See also Figure S3.

Because *kri-1* functions independently of *cep-1* and impinges on the core death pathway, we were interested to know whether *kri-1* is cooperating with other genes known to regulate germ cell death independently or downstream of *cep-1*. In particular, the histone deacetylase *sir-2.1* [19], the MAP kinase *pmk-3* [20], and the retinoblastoma (RB) ortholog *lin-35* [21] have all been shown to regulate germ cell death independently of *cep-1*. In addition to activating cell death independently of *cep-1* in a manner similar to *kri-1*, the SIR-2.1 protein exits the nuclei of germ cells after DNA damage [19]. To determine whether the relocalization of SIR-2.1 is required for *kri-1*-mediated germ cell death, we immunostained *kri-1(0)* animals with SIR-2.1 antibodies to ascertain whether SIR-2.1 protein levels or localization was altered. Although we found that *kri-1* did not affect the SIR-2.1 protein staining pattern (Figure 3A), it still remained possible that *kri-1* and *sir-2.1* function in the same pathway. To address this, we created a double heterozygous mutant containing both the *kri-1(0)* and *sir-2.1(lf)* mutations (*kri-1(0)*/+; *sir-2.1(lf)*/+), and we observed wild-type levels of germ cell apoptosis in response to DNA damage (data not shown), suggesting that these genes operate in different pathways. In contrast to *sir-2.1* and *kri-1*, which positively regulate germline apoptosis, the MAP kinase gene *pmk-3* inhibits germline apoptosis independently of *cep-1* [20]. Therefore, we tested whether *kri-1* was required for germ cell death caused by loss of function of *pmk-3*. We created *kri-1(0); pmk-3(lf)* double mutants and found that germ cell death was suppressed to the same degree as *kri-1(0)* single mutants (Figure 3B), suggesting that *kri-1* is epistatic to *pmk-3* and does not regulate cell death through *pmk-3*. We do not believe that *pmk-3* regulates *kri-1* because *kri-1* transcript levels and protein localization remained unchanged in *pmk-3(lf)* mutants (Figure S3). Finally, because *lin-35* positively regulates germ cell apoptosis by controlling the levels of the CED-9 protein (i.e., loss of *lin-35* lead to an increase in CED-9 protein levels) [21], we tested whether *kri-1* functions through *lin-35*/RB by quantifying CED-9 protein levels in *kri-1(0)* animals by western blot. We found that CED-9 protein levels were unaffected in *kri-1(0)* (A. Ross, personal communication; data not shown), suggesting that *kri-1* does not regulate germline apoptosis through this pathway.

Additionally, it has been shown that *kri-1* influences the localization of the forkhead transcription factor DAF-16 in the intestine by responding to signals from the germline and regulating worm life span [16] and that DAF-16 may negatively regulate IR-induced germ cell apoptosis [17]. These two pieces of evidence suggested that *kri-1* might function through *daf-16* to regulate germ cell death. Animals fed *daf-16(RNAi)* exhibited wild-type levels of germ cell death in response to IR (Figure 3C), consistent with published results reporting that DAF-16 has a weak effect on germ cell death [17, 19]. We tested whether *kri-1(0)* could suppress apoptosis in animals fed *daf-16(RNAi)* and found that it did (Figure 3C), which we confirmed by creating *kri-1(0) daf-16(lf)* double mutants (data not shown). This suggests that *kri-1* does not require *daf-16* to mediate its apoptotic function. Alternatively, it was possible that *daf-16* regulates *kri-1* to mediate germ cell death; however, neither *kri-1* transcript nor protein levels were significantly affected in *daf-16(lf)* mutants (Figure S3). These observations reveal that *kri-1* is involved in a novel pathway that regulates germ cell death in response to DNA damage.

**Figure 4:**
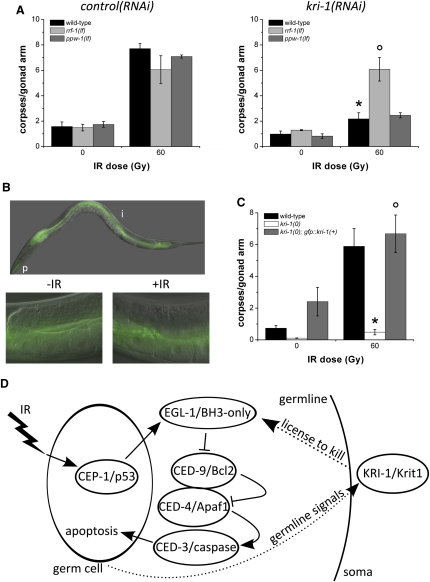
*kri-1* Regulates Germ Cell Death from Somatic Tissues by a Cell-Nonautonomous Mechanism (A) Germ cell death was quantified after treatment with IR in wild-type (black), *rrf-1(lf)* (light gray), and *ppw-1(lf)* (dark gray) L4 worms fed bacteria producing double-stranded RNA against a control gene (left panel) or *kri-1* (right panel). Data represent mean ± SEM of three independent experiments and at least 35 germlines in total per strain per condition. ^∗^p < 0.01 versus *control(RNAi)*; °p < 0.01 versus *kri-1(RNAi)*. (B) GFP::KRI-1 expressed under the control of the endogenous *kri-1* promoter is detectable in the pharynx (p) and intestine (i) of transgenic animals (top panel). GFP::KRI-1 is excluded from the germline in unirradiated animals (bottom left panel) and does not change localization after irradiation (bottom right panel). Representative images of at least three independent experiments are shown. (C) Apoptotic germ cells were quantified in wild-type (black), *kri-1(0)* (white), and a *kri-1(0)* strain expressing a wild-type copy of GFP::KRI-1 in the soma (dark gray). Data represent mean ± SEM of three independent experiments and at least 40 germlines in total per strain per condition. ^∗^p < 0.01 versus wild-type; °p < 0.01 versus *kri-1(0)*. (D) Model depicting somatic requirement for *kri-1* in promoting germ cell death in response to DNA damage. We hypothesize that there are “license to kill” factors secreted from the soma into the germline to mediate cell death. Solid lines represent known regulatory interactions; dotted lines represent hypothetical interactions.

There are two possible mechanisms by which *kri-1* may promote germ cell death. The first is a cell-autonomous mechanism, in which *kri-1* regulates the core death pathway (EGL-1 or CED-9) in germ cells to initiate cell death. Alternatively, it is possible that *kri-1* regulates cell death outside of germ cells (i.e., from somatic cells) via a novel pathway. In support of the latter hypothesis, *kri-1* is required to extend the life span of worms through its effects on DAF-16 in the intestine, possibly by receiving signals from germ cells [16]; in addition, microarray data suggest that *kri-1* is not expressed in the germline [22]. To distinguish between these possibilities, we took advantage of tissue-specific RNAi in *C. elegans* and selectively knocked down *kri-1* in germ cells and the soma in *rrf-1(lf)* [23] and *ppw-1(lf)* [24] mutants, respectively, and quantified IR-induced germ cell apoptosis [21] (Figure 4A). Wild-type, *rrf-1(lf)*, and *ppw-1(lf)* mutants fed bacteria producing *control(RNAi)* had similar numbers of germ cell corpses after DNA damage. Ablation of *kri-1* by RNAi in wild-type animals inhibited DNA damage-induced germ cell apoptosis to the same extent as *kri-1(0)* mutants. However, selective knockdown of *kri-1* in germ cells in *rrf-1(lf)* mutants caused an increase in IR-induced apoptosis, suggesting that *kri-1* expression in germ cells is not required to promote apoptosis. Conversely, specific knockdown of *kri-1* in the soma in *ppw-1(lf)* mutants prevented germ cell death, suggesting that *kri-1* is required in somatic tissue to regulate germ cell death. In support of this contention, we were able to rescue damage-induced germ cell apoptosis to wild-type levels by expressing GFP::KRI-1 from a somatic extrachromosomal array (Figures 4B and 4C). Although it is possible that low-level expression of GFP::KRI-1 in the germline may account for this observation, the fact that extrachromosomal arrays are generally silenced in the *C. elegans* germline [25] strongly supports a model in which *kri-1* is required in nondying somatic cells to promote germ cell death.

Collectively, these data imply a novel mechanism whereby somatic cells communicate with germ cells to promote their death in response to DNA damage (Figure 4D). Indeed, other genes have been shown to participate in germline-soma signaling during proliferation and differentiation of the germline [26, 27], dauer formation, and life-span control [28, 29], confirming that these two tissues can signal to each other in response to certain stimuli. The fact that *kri-1*/*KRIT1* regulates germ cell death from cells outside of the germline independently of *cep-1*/*p53* implies that cells not fated to die (somatic cells) in *C. elegans* can regulate the core death pathway in germ cells by novel, cell-nonautonomous mechanisms. In the case of *kri-1*, there are several ways in which it may be performing this function. First, we examined the possibility that *kri-1* may be acting through *daf-9* and *daf-12* to promote apoptosis, in a manner analogous to its proposed role in life-span control [16], by feeding *kri-1(RNAi)* to *daf-9(lf)* and *daf-12(lf)* mutants. We found that both mutants exhibited a resistance to apoptosis when fed *kri-1(RNAi)*, suggesting that *kri-1* does not act through these two genes (Figure S4). Second, although we have shown that *kri-1* does not affect the transcript levels of the BH3-only gene *egl-1*, it is possible that the *kri-1* is required to activate the EGL-1 protein. Similar to mammalian BH3-only proteins, EGL-1 may require other coactivating proteins or modifications in order to induce cell death in germ cells. For example, BID, BAD, and BIM/BMF are proapoptotic BH3-only proteins regulated at the posttranslational level through cleavage, phosphorylation, and sequestration by interacting proteins, respectively [30]. Therefore, it is formally possible that KRI-1 may facilitate the activation of EGL-1 by similar transcription-independent mechanisms. Alternatively, KRI-1 may be involved in receiving signals from the germ cells, which results in the subsequent release of death-inducing factors. Although further studies are required to resolve the biochemical mechanism by which KRI-1 dictates germ cell death from the soma, our observations reveal a novel cross-tissue signaling mechanism whereby somatic tissue can promote germ cell death in response to DNA damage in *C. elegans*, which may have broader applicability to cell death in general.

## Supplemental Information

Document S1. Four Figures and Supplemental Experimental Procedures

Document S2. One Table

## References

[1] Ellis H.M., Horvitz H.R. (1986). Genetic control of programmed cell death in the nematode C. elegans. Cell.

[2] Hipfner D.R., Cohen S.M. (2004). Connecting proliferation and apoptosis in development and disease. Nat. Rev. Mol. Cell Biol..

[3] Ahmed S., Hodgkin J. (2000). MRT-2 checkpoint protein is required for germline immortality and telomere replication in C. elegans. Nature.

[4] Gartner A., Milstein S., Ahmed S., Hodgkin J., Hengartner M.O. (2000). A conserved checkpoint pathway mediates DNA damage-induced apoptosis and cell cycle arrest in C. elegans. Mol. Cell.

[5] Hofmann E.R., Milstein S., Boulton S.J., Ye M., Hofmann J.J., Stergiou L., Gartner A., Vidal M., Hengartner M.O. (2002). Caenorhabditis elegans HUS-1 is a DNA damage checkpoint protein required for genome stability and EGL-1-mediated apoptosis. Curr. Biol..

[6] Ahmed S., Alpi A., Hengartner M.O., Gartner A. (2001). C. elegans RAD-5/CLK-2 defines a new DNA damage checkpoint protein. Curr. Biol..

[7] Derry W.B., Putzke A.P., Rothman J.H. (2001). Caenorhabditis elegans p53: Role in apoptosis, meiosis, and stress resistance. Science.

[8] Schumacher B., Hofmann K., Boulton S., Gartner A. (2001). The C. elegans homolog of the p53 tumor suppressor is required for DNA damage-induced apoptosis. Curr. Biol..

[9] Yuan J.Y., Horvitz H.R. (1990). The Caenorhabditis elegans genes ced-3 and ced-4 act cell autonomously to cause programmed cell death. Dev. Biol..

[10] Conradt B., Horvitz H.R. (1998). The C. elegans protein EGL-1 is required for programmed cell death and interacts with the Bcl-2-like protein CED-9. Cell.

[11] del Peso L., González V.M., Núñez G. (1998). Caenorhabditis elegans EGL-1 disrupts the interaction of CED-9 with CED-4 and promotes CED-3 activation. J. Biol. Chem..

[12] Chen F., Hersh B.M., Conradt B., Zhou Z., Riemer D., Gruenbaum Y., Horvitz H.R. (2000). Translocation of C. elegans CED-4 to nuclear membranes during programmed cell death. Science.

[13] Schumacher B., Hanazawa M., Lee M.H., Nayak S., Volkmann K., Hofmann E.R., Hengartner M., Schedl T., Gartner A. (2005). Translational repression of C. elegans p53 by GLD-1 regulates DNA damage-induced apoptosis. Cell.

[14] Sahoo T., Johnson E.W., Thomas J.W., Kuehl P.M., Jones T.L., Dokken C.G., Touchman J.W., Gallione C.J., Lee-Lin S.Q., Kosofsky B. (1999). Mutations in the gene encoding KRIT1, a Krev-1/rap1a binding protein, cause cerebral cavernous malformations (CCM1). Hum. Mol. Genet..

[15] Laberge-le Couteulx S., Jung H.H., Labauge P., Houtteville J.P., Lescoat C., Cecillon M., Marechal E., Joutel A., Bach J.F., Tournier-Lasserve E. (1999). Truncating mutations in CCM1, encoding KRIT1, cause hereditary cavernous angiomas. Nat. Genet..

[16] Berman J.R., Kenyon C. (2006). Germ-cell loss extends C. elegans life span through regulation of DAF-16 by kri-1 and lipophilic-hormone signaling. Cell.

[17] Quevedo C., Kaplan D.R., Derry W.B. (2007). AKT-1 regulates DNA-damage-induced germline apoptosis in C. elegans. Curr. Biol..

[18] Schumacher B., Schertel C., Wittenburg N., Tuck S., Mitani S., Gartner A., Conradt B., Shaham S. (2005). C. elegans ced-13 can promote apoptosis and is induced in response to DNA damage. Cell Death Differ..

[19] Greiss S., Hall J., Ahmed S., Gartner A. (2008). C. elegans SIR-2.1 translocation is linked to a proapoptotic pathway parallel to cep-1/p53 during DNA damage-induced apoptosis. Genes Dev..

[20] Lettre G., Kritikou E.A., Jaeggi M., Calixto A., Fraser A.G., Kamath R.S., Ahringer J., Hengartner M.O. (2004). Genome-wide RNAi identifies p53-dependent and -independent regulators of germ cell apoptosis in C. elegans. Cell Death Differ..

[21] Schertel C., Conradt B. (2007). C. elegans orthologs of components of the RB tumor suppressor complex have distinct pro-apoptotic functions. Development.

[22] Reinke V., Smith H.E., Nance J., Wang J., Van Doren C., Begley R., Jones S.J., Davis E.B., Scherer S., Ward S., Kim S.K. (2000). A global profile of germline gene expression in C. elegans. Mol. Cell.

[23] Sijen T., Fleenor J., Simmer F., Thijssen K.L., Parrish S., Timmons L., Plasterk R.H., Fire A. (2001). On the role of RNA amplification in dsRNA-triggered gene silencing. Cell.

[24] Tijsterman M., Okihara K.L., Thijssen K., Plasterk R.H.A. (2002). PPW-1, a PAZ/PIWI protein required for efficient germline RNAi, is defective in a natural isolate of C. elegans. Curr. Biol..

[25] Kelly W.G., Xu S., Montgomery M.K., Fire A. (1997). Distinct requirements for somatic and germline expression of a generally expressed Caernorhabditis elegans gene. Genetics.

[26] Killian D.J., Hubbard E.J.A. (2004). C. elegans pro-1 activity is required for soma/germline interactions that influence proliferation and differentiation in the germ line. Development.

[27] Kimble J.E., White J.G. (1981). On the control of germ cell development in Caenorhabditis elegans. Dev. Biol..

[28] Apfeld J., Kenyon C. (1998). Cell nonautonomy of C. elegans daf-2 function in the regulation of diapause and life span. Cell.

[29] Wolkow C.A., Kimura K.D., Lee M.S., Ruvkun G. (2000). Regulation of C. elegans life-span by insulinlike signaling in the nervous system. Science.

[30] Puthalakath H., Strasser A. (2002). Keeping killers on a tight leash: Transcriptional and post-translational control of the pro-apoptotic activity of BH3-only proteins. Cell Death Differ..

