# Supplementary material for: Cell-Nonautonomous Regulation of C. elegans Germ Cell Death by kri-1
Source: Curr Biol. 2010 Feb 23;20(4):333–8. doi: 10.1016/j.cub.2009.12.032 (PMC2829125; doi:10.1016/j.cub.2009.12.032)
Supplement: Document S1. Four Figures and Supplemental Experimental Procedures [file mmc1.pdf]

Current Biology, Volume *20*

Supplemental Information

Cell-Nonautonomous Regulation

of *C. elegans* Germ Cell Death by *kri-1*

Shu Ito, Sebastian Greiss, Anton Gartner, and W. Brent Derry

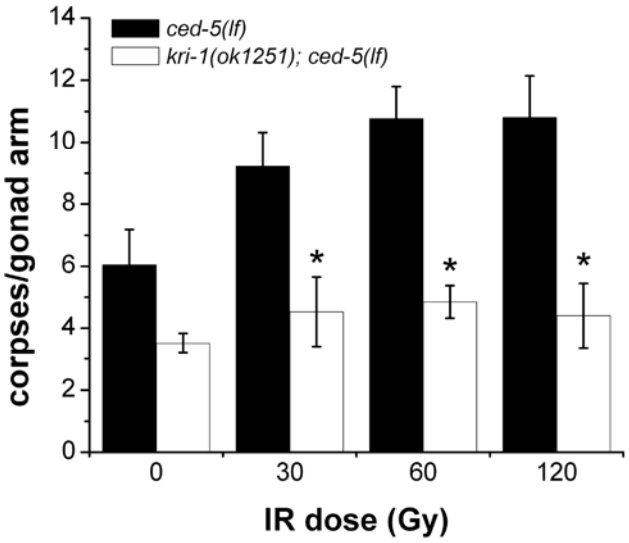

Figure S1. *kri-1* Is Required for Germ Cell Death in Response to IR

To increase the sensitivity of the apoptosis assay, *kri-1(ok1251)* mutants were crossed into the engulfment defective mutant *ced-5(lf)* strain in which germ cell corpses persist and consequently exhibit increased numbers of corpses. Germ cell death was quantified 24 hours after DNA damage of synchronized young adult worms. The dose dependent increase in germ cell corpses observed in *ced-5(lf)* single mutants (black bars) is suppressed in the presence of the *kri-1* mutation in the *kri-1(ok1251); ced-5(lf)* double mutant (white bars), further suggesting that *kri-1* is required for cell death after DNA damage. Data represent mean ± s.e.m. of three independent experiments and at least 40 germlines in total per strain per condition. *$p < 0.05$ when compared to wild-type worms.
